# Origin of continental crust on early Earth

**DOI:** 10.1093/nsr/nwaf341

**Published:** 2025-08-18

**Authors:** Yong-Fei Zheng

**Affiliations:** State Key Laboratory of Lithospheric and Environmental Coevolution, School of Earth and Space Sciences, University of Science and Technology of China, China

## Abstract

The nature of tectonic realms is a key to the origin of continental crust on early Earth. However, it is questionable to infer the composition of major elements in crustal rocks only from the composition of trace elements in zircon. Whereas non-plate tectonics was popular in generating the mafic crust in the Hadean, the operation of plate tectonics is necessary in transforming the mafic crust to the felsic crust in the Archean.

The origin of continental crust on early Earth (the Hadean and Archean eons) is contentious, with the debate mainly between mechanisms of plate and non-plate tectonics [1,2]. Although both contentions find circumstantial support from natural observations and numerical simulations, it is generally agreed that the early continental crust was predominated by felsic lithologies such as tonalite–trondhjemite–granodiorite (TTG) suites, whose precursor was a kind of mafic lithology such as oceanic basalts. However, it is controversial what type of basaltic rocks (oceanic plateau vs mid-ocean ridge) underwent partial melting to produce TTG magmas. Furthermore, the proportion of mafic and felsic lithologies is significantly different between the Hadean and Archean eons. Whereas the Hadean crust was dominated by mafic lithology [3], the Archean crust was predominated by TTG suites with minor amounts of greenstones and potassic granitoids [1,2]. Such a difference in the composition of major elements has often been overlooked in architecting the composition of continental crust from zircon geochemistry [4,5], leading to a new debate in its lithochemistry between andesitic [4] and non-andesitic [5]. In this regard, it is critical to examine whether the geochemical signatures retrieved from zircon can be used to infer the composition of bulk rocks on early Earth.

The operation of plate tectonics on modern Earth has generated the mafic crust in such lithologies as mid-ocean ridge basalts, oceanic arc basalts and continental arc andesites along plate margins [6]. Partial melting of these mafic lithologies gives rise to the continental crust of felsic lithologies with mafic residues. Although such two-stage processes are common in building the continental crust on modern Earth, they are not certain in the origin of continental crust on early Earth. On the one hand, the mafic crust can be generated in different ways, through either fractional crystallization of metal from an ultramafic magma ocean during the core formation in the Hadean [7], or partial melting of ultramafic mantle lithology via either mantle plume magmatism in the realm of a stagnant lid or rifting magmatism in the realm of mobile lids [2]. On the other hand, the felsic continental crust can be produced by partial melting of the mafic crust either in oceanic plateaux [8] or along convergent plate margins [2,5,9,10]. In either case, it is critical to examine whether the continental crust would acquire its distinct geochemical signatures during mafic or felsic magmatism.

The composition of trace elements in detrital zircon of Hadean U–Pb ages has been used to argue for or against the operation of plate tectonics on early Earth [4,5]. In doing so, it generally assumes that continental crust is characterized by arc-like trace element signatures, such as depletion in compatible Nb and Ta but enrichment in incompatible large ion lithophile elements (LILE), Pb and light rare Earth elements (LREE) relative to compatible heavy rare Earth elements (HREE), and thus was originally generated by subduction zone magmatism [2]. However, this assumption is challenged by Turner *et al.* [7] through modelling the formation of the Hadean mafic protocrust from the magma ocean to architect its trace element composition similar to the both average Archean and modern continental crust. If this model could be validated, it would reshape debates on tectonic realm, crustal growth and zircon genesis on early Earth. Unfortunately, it overlooks the significant difference in melt-mobile incompatible LILE and LREE abundances between Archean and Phanerozoic mid-ocean ridge basalts, a geochemical feature of secular LILE and LREE depletion due to continuous low-degree extraction of hydrous felsic melts from subducting oceanic slabs since the operation of plate tectonics in the Archean [2]. Even if the arc-like signatures could be completely transferred from the Hadean mafic protocrust into the early continental crust without modification, appropriate physicochemical conditions would be necessary to produce felsic TTG magmas with high Sr/Y and La/Yb ratios. Furthermore, it is highly questionable whether no geochemical differentiation between melt-mobile incompatible and melt-immobile compatible trace elements would considerably take place during the crustal anatexis. Additionally, it is problematic to suppose that the trace element composition of the mafic protocrust on early Earth would resemble oceanic arc basalts rather than mid-ocean ridge basalts on modern Earth.

It is known from experimental petrology that felsic TTG magmas cannot be formed from the mantle through either fractional crystallization of the ultramafic magma ocean or partial melting of the ultramafic lithology [6]. Therefore, the mafic crust is the most possible precursor to generate the felsic crust. As such, the TTG-like geochemical signatures in early continental crust would be derived from partial melting of the hydrated, ultrathick mafic crust in the stability fields of both garnet and rutile at convergent plate margins [11]. This is achieved by three-stage processes in ancient Wilson cycles in order to form the continental crust on early Earth (Fig. 1). At first, the juvenile, thick mafic crust would be hydrated due to seawater-hydrothermal alteration of the oceanic crust during its generation at mid-ocean ridges through seafloor spreading (Fig. 1A). The hydrated, thick mafic crust was then transported to convergent microplate margins, where it was further thickened by collisional compression (Fig. 1B). Finally, the ultrathick, hydrated mafic crust underwent partial melting due to upwelling of the asthenospheric mantle in response to foundering of the lithospheric mantle through either gravitational delamination or convective erosion (Fig. 1C), giving rise to felsic TTG magmas. This TTG petrogenetic model is testified not only by the machine learning study of zircon geochemistry [5] but also by two combined studies of whole-rock K and zircon O isotope compositions [9,10], arguing for the operation of plate tectonics on early Earth. Although the TTG magmatism was produced at convergent plate margins, the crustal anatexis would take place inside the collisionally thickened mafic crust rather than the subducted oceanic slab [2].

**Figure 1.**
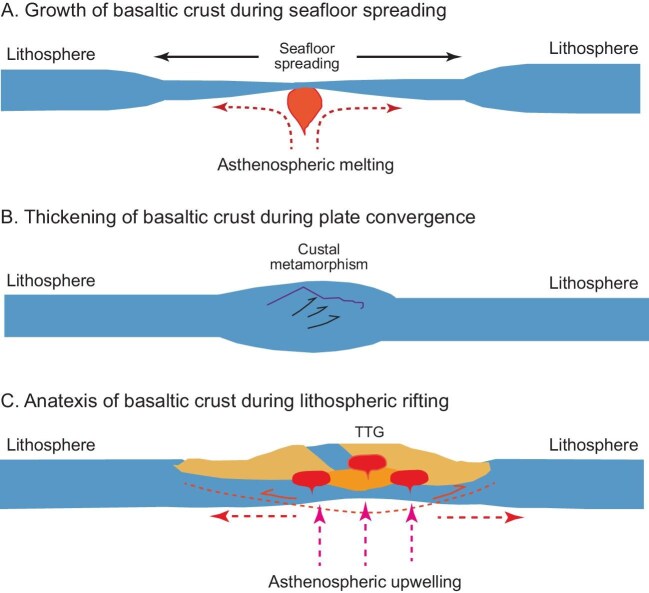
Schematic diagram for the generation of Archean TTG magmas in a Wilson cycle on early Earth (revised from Zheng *et al.* [11]). (A) Hydration of basaltic rocks at mid-ocean ridges during seafloor spreading. (B) Collisional thickening of the thick oceanic crust during plate convergence. (C) Lithospheric rifting due to upwelling of the asthenospheric mantle induced by foundering of the overlying lithospheric mantle, giving rise to TTG magmas via partial melting of the hydrated, ultrathick and basaltic crust along converged plate margins.

Traditionally, a Wilson cycle was considered to comprise three stages: seafloor spreading; oceanic subduction; and continental collision. In reality, a fourth stage is necessary to develop the continental breakup from the continental collision zone [11]. This is only achieved by mantle poloidal convection in the form of

either mantle plume from the lower mantle or asthenospheric upwelling from the upper mantle consequential to lithospheric foundering via gravitational delamination or convection erosion [6]. Furthermore, the oceanic crust was much thicker in the Archean than in the Phanerozoic [2], tending to collisional thickening instead of subduction stacking during plate convergence. Therefore, felsic TTG magmas would be preferentially produced in oceanic collisional zones rather than in oceanic subduction zones. As such, these four-stage processes together constitute a complete Wilson cycle [11], witnessing the operation of ancient plate tectonics in the Archean.

The debate in the origin of continental crust between the mechanisms of plate and non-plate tectonics on early Earth has great bearing not only on the nature of tectonic realms but also on the composition of continental crust [1,2]. The mafic protocrust was popular in the Hadean [3,7], and its localized anatexis is necessary in order to make felsic magmas for zircon growth [12]. It is highly possible that the Hadean protocrust was generated in the setting of non-plate tectonics [7], with its partial melting to form peritectic, anatectic and magmatic zircons [12]. After entering the Archean, the felsic TTG rocks would become more and more popular, witnessing the operation of microplate tectonics in coupled divergent–convergent systems [2,6]. Although various tectonic mechanisms have been suggested for the production of Archean TTG magmas [1,2], there is no substantial difference, not only between mantle plume and hot pipe in bottom-up processes except the spatial scale (large vs small) but also between delamination and sagduction in top-down processes except the timescale (short vs long).

Zircon geochemistry is an important means in deciphering the composition of continental crust [12]. However, the problem has been encountered in using zircon trace element composition to deduce the major element composition of continental crust on early Earth, typically yielding either andesitic or non-andesitic lithochemistry [4,5]. Therefore, it merits a reminder of the problem in the methodology of applying zircon geochemistry to the reconstruction of crustal lithochemistry. In this regard, it is critical to adopt the partition coefficients for given trace elements between zircon and melt. So too in building the Hadean mafic protocrust from the magma ocean with partition coefficients for those trace elements between silicate melt and minerals [7]. Furthermore, with respect to zircon growth during magmatism, it is necessary for magmas to achieve zirconium saturation during geological processes such as partial melting and fractional crystallization [12]. In this regard, it is critical to determine whether Hadean zircons would grow from primary mafic magmas or secondary felsic magmas. Furthermore, there are variable elevations of Archean zircon O isotope ratios relative to the normal mantle value [8–10], making the low-temperature seawater-hydrothermally altered mid-ocean ridge basalts (generated during seafloor spreading along divergent plate margins) the best candidate for the parental rocks of growing zircon during their partial melting and the fractional crystallization of their derivative magmas [2]. This argument has also gained strong support from whole-rock K isotope studies [9,10]. So far, no significant elevation of the zircon O isotope ratios has been reported for oceanic plateau basalts, making the mantle plume–sagduction hypothesis [8] less possible.

In summary, the processes of plate tectonics would play a significant role in the formation of both mafic and felsic crust in the Archean, and the dominant mechanism for the TTG petrogenesis involves the partial melting of the hydrated ultrathick basaltic crust at convergent plate margins. In contrast, the mafic protocrust in the Hadean was generated in the realm of non-plate tectonics, and it is not necessary to hypothesize its anatexis in the realm of plate tectonics for the formation of felsic magmas and thus for zircon growth. It is important to note that the continental crust on early Earth was produced above a hotter, more energetic mantle with no considerable depletion or enrichment in melt-mobile incompatible trace elements [2]. Although Hadean zircons provide considerable insights into the conditions of geological processes such as depths of either fractional crystallization or partial melting on early Earth, they are inherently limited in architecting the crustal composition of major elements. Therefore, it is critical to distinguish in the origin of distinct geochemical signatures for continental crust between magma source and crust-forming process. As such, caution must be exercised when linking zircon trace element data to bulk rock chemistry in deciphering the origin of early continental crust.
